# 3-Step Combined Technique for Correction of Involutional Lower Eyelid Ectropion: A Case Series

**DOI:** 10.3390/jcm14010128

**Published:** 2024-12-29

**Authors:** Corrado Rubino, Emilio Trignano, Stefano Dore, Antonio Pinna, Nicola Tsatsaris, Federico Ziani, Lucia Sangalli, Matilde Tettamanzi, Silvia Rampazzo

**Affiliations:** 1 Department of Medicine, Surgery and Pharmacy, University of Sassari, 07100 Sassari, Italy; corubino@uniss.it (C.R.); etrignano@uniss.it (E.T.); stdore@uniss.it (S.D.); apinna@uniss.it (A.P.); 2 Plastic Surgery Unit, University Hospital Trust of Sassari, 07100 Sassari, Italy; zianifederico@gmail.com (F.Z.); l.sangalli@studenti.uniss.it (L.S.); m.tettamanzi@studenti.uniss.it (M.T.); 3 Ophthalmology Unit, University Hospital Trust of Sassari, 07100 Sassari, Italy; 4 Section of Neurosurgery, Department of Neurosciences, Biomedicine and Movement Sciences, University Hospital, 37126 Verona, Italy; nicolatsatsaris@gmail.com; 5 Plastic, Reconstructive and Aesthetic Surgery Training Program, University of Sassari, 07100 Sassari, Italy

**Keywords:** involutional ectropion, lower eyelid malposition, wedge resection, combined procedure

## Abstract

**Background:** Involutional lower eyelid ectropion is a common disorder of the elderly population. Several surgical approaches have been described in the literature to address the multifactorial nature of this condition, each targeting different factors contributing to its development. Nevertheless, no single procedure has proven to be superior to the others. This study aims to assess the safety and effectiveness of a new 3-step combined technique in treating involutional ectropion. **Methods:** The surgical technique consists of a combination of lateral lid-shortening with removal of a base-up triangle (modified Bick procedure), lower eyelid skin release, and transposition of a laterally based myocutaneous flap from the upper eyelid. A retrospective chart review was conducted for all patients treated with this procedure at our institution between 2012 and 2023 was performed. Self-reported patient satisfaction with functional and esthetic outcomes was evaluated three months after surgery. **Results:** A total of thirty-six patients (forty-five eyelids) were included in the study, with a mean follow-up period of 22 months (range 3–144). Minor surgical revision was performed in one case for early wound dehiscence following premature stitches removal. The overall success rate was 93.2%, with one case of undercorrection and two cases of recurrence recorded at three years postoperatively. No additional complications or recurrences were observed during the follow-up period. **Conclusions:** The 3-step combined procedure demonstrated high efficacy and safety, offering excellent functional and esthetic outcomes. This approach provides a reliable solution for treating involutional ectropion, making it a valuable addition to the surgical options for this condition.

## 1. Introduction

Involutional lower eyelid ectropion refers to an outward turning of the eyelid margin caused by age-related degeneration of the structural and functional components of the eyelid. This condition is one of the most common types of eyelid malposition and significantly impacts the quality of life of affected individuals, particularly older adults. Studies estimate a prevalence of approximately 2.9% among individuals aged 60 years or older, with the incidence increasing markedly with age [1]. The condition not only causes functional impairments such as chronic epiphora, ocular irritation, and visual discomfort but also results in esthetic concerns that can affect psychological well-being. Additionally, ectropion increases the risk of exposure keratopathy, conjunctival inflammation, and recurrent infections, highlighting the clinical importance of its timely diagnosis and management.

Lower eyelid involutional malposition is a multifactorial condition usually resulting from a combination of eyelid laxity and mechanical imbalance between the anterior and posterior lamellae. The horizontal and vertical instability of the lower eyelid, resulting from tarsal ligament laxity, loosened tarsus, and disinsertion or weakening of the lower eyelid retractors [2,3,4,5], represents the main aetiologic event in the development of lower lid malposition. These age-related structural changes, in fact, render the lower eyelid prone to rotate either inward or outward [6]. A key contributor to this imbalance is the degeneration or atrophy of the orbicularis oculi muscle (OOM), which loses its ability to counteract the gravitational force pulling the eyelid outward. This dysfunction creates a disparity in the interaction of the anterior and posterior lamellae, leading to the eversion of the lid margin. When the lid margin begins to rotate outward, the conjunctiva is exposed and the punctum becomes everted, resulting in symptoms such as excessive tearing, ocular discomfort, and irritation. Over time, inflammatory changes occur, leading to secondary thickening of the tarsus and contraction of the skin of the lower eyelid, which mechanically exacerbates the ectropion [3,7]. Additionally, age-related anatomical changes, including the descent of malar fat pad and midface volume loss result in the loss of tissue support of the lower eyelid, also contribute to pulling the lower eyelid down [8]. These factors collectively cause malalignment of the eyelid margin, eversion of the punctum, and conjunctival exposure, which leads to excessive tearing and secondary inflammation of ocular tissues. Prolonged exposure may result in fibrosis and scarring, perpetuating the lid malposition. This cascade of events underscores the complex interplay of factors contributing to involutional ectropion.

Various procedures [8,9,10,11,12,13,14,15,16] targeting different aetiologic factors have been described for the correction of involutional ectropion, but no ideal single technique has been agreed upon. The aim of this paper is to assess the safety and effectiveness of a new combined procedure in treating involutional ectropion. The technique consists of a combination of three steps: (1) lateral lid-shortening with removal of a base-up triangle (modified Bick procedure [9]) to address the instability of the eyelid, (2) lower eyelid skin release to reduce the outpulling vector, and (3) the transposition of a laterally based myocutaneous flap from the upper lid to stabilize the eyelid position. To the best of our knowledge, the combination of these three procedures has not been described in the literature before for the treatment of lower lid involutional ectropion and may offer a comprehensive solution for addressing the causative factors involved in the development of this condition.

## 2. Materials and Methods

This retrospective study analyzed the medical records of the patients who underwent lower lid involutional ectropion correction with the 3-step technique at the Plastic Surgery Unit, Azienda Ospedaliera-Universitaria di Sassari, Sassari, Italy, between 2012 and 2023. Surgeries were performed by a senior surgeon and a junior surgeon.

The inclusion criterion for this study was the diagnosis of involutional lower eyelid ectropion, defined as a symptomatic outward rotation of the lower eyelid associated with lid laxity. Patients exhibiting a history of previous trauma or surgery to the periorbital region, as well as those with ectropion caused by other etiologies such as congenital, cicatricial, paralytic, or spasmodic factors, were excluded from the study to ensure a homogenous population. Comprehensive demographic data, including patient age and gender, were systematically recorded alongside baseline assessments of the severity of the lower lid ectropion and associated symptoms reported by the patients. The severity of ectropion was categorized into three grades: grade I involved lower eyelid eversion with underlying scleral exposure (scleral show); grade II was characterized by lower eyelid eversion with visible lower conjunctiva; and grade III represented severe cases with exposure of the fornix. Horizontal lid laxity was evaluated through a measurement of the lower eyelid’s distraction from the globe, with a distance greater than 6 mm deemed abnormal and indicative of significant laxity. Additionally, the orbicularis oculi muscle tone and lid laxity were further assessed using the snapback test, where any delay in the lower lid returning to its original position after being pulled away from the globe was classified as a positive finding. Data regarding post operative complications, such as infection, hematoma, and wound dehiscence were recorded. To ensure the reliability of outcomes, a minimum follow-up period of three months was required for inclusion in the study. The primary outcome measures of this investigation encompassed anatomical, esthetic, and functional success. Surgical success was defined as the restoration of the proper apposition of the lower eyelid to the ocular surface, combined with the resolution of symptoms previously associated with ectropion. Cases of undercorrection or overcorrection were identified by the presence of eyelid malposition observed during the three-month follow-up or earlier. In contrast, recurrence of ectropion was defined as the reappearance of the outward eyelid rotation beyond the three-month postoperative period. Esthetic and functional outcomes were evaluated based on patients’ self-reported satisfaction at three months’ follow-up.

### 2.1. Surgical Technique

Pre-operative markings were meticulously made with the patient positioned upright to ensure proper alignment and optimal surgical planning. In the lower eyelid, a horizontal line was drawn approximately 2 mm below the eyelid margin, serving as a guide for subsequent incisions. For the upper eyelid, a laterally based myocutaneous flap was delineated, with the inferior incision line placed in the supratarsal fold about 8 mm from the ciliary margin. This distance was adjusted according to the patient’s specific anatomy to ensure a natural postoperative appearance. The incision extended laterally to the canthal area, seamlessly joining the lower eyelid incision line to create a continuous surgical field. The superior incision line of the flap, as well as its overall width, was determined using the Pinch technique [17], which allowed for precise measurement of redundant tissue to be excised and avoided excessive tightening in the upper eyelid.

Local anesthesia was administered to ensure patient comfort and a bloodless surgical field. A mixture of 2% lidocaine and 1:100,000 epinephrine was injected using a fine 25-gauge needle. The anesthesia was applied in a targeted manner, involving the superior and inferior eyelids, the lateral canthal angle, and the subconjunctival region of the lower eyelid fornix. This comprehensive approach ensured adequate numbness and minimized intraoperative discomfort.

**Figure 1:**
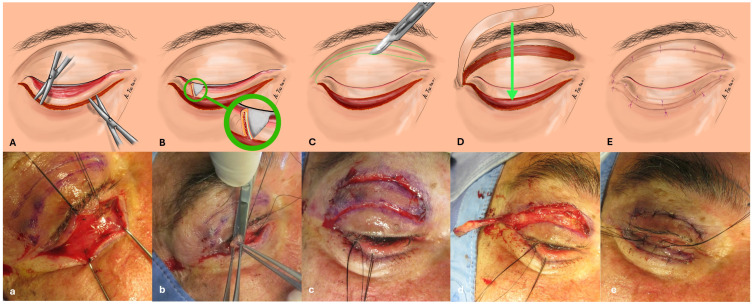
The illustration above provides a graphic representation, and the photograph below captures an intraoperative view of the 3-step surgical technique designed for the treatment of involutional lower eyelid ectropion. This comprehensive approach is depicted step by step as follows: (**A**,**a**) Initiation with a lower eyelid skin incision followed by the lysis of adhesions between skin and OOM; (**B**,**b**) Execution of a lateral lid shortening via the resection of a full thickness, base-up triangle. An enlarged view of the triangular resection is drawn within the green circle; (**C**,**c**) Harvesting a laterally based orbicularis oculi myocutaneous flap from the upper eyelid; (**D**,**d**) Transposition of the harvested myocutaneous flap to the recipient site (the green line indicates the direction of the transposition); (**E**,**e**) Skin closure.

A horizontal incision was made with blade no. 15 along the previously marked line in the lower eyelid. The incision was carried through the subcutaneous plane. Subsequently, adhesions between the skin and the orbicularis oculi muscle were lysed along the entire length of the eyelid using Wescott scissors, facilitating smooth tissue manipulation (Figure 1a). To address horizontal laxity, the lower eyelid was shortened by performing a base-up, full-thickness triangular resection of tissue at the lateral canthus (Figure 1b). The width of the resected triangle was carefully tailored based on the degree of laxity observed. After making the initial full-thickness lateral incision, approximately 2 mm from the lateral canthal corner, the eyelid was gently distracted laterally to assess the exact amount of tissue that needed to be excised. Following resection, meticulous closure of the tarsus was performed. A continuous running suture of Nylon 5-0 was performed passing through the deep portion of the tarsus, ensuring structural integrity and proper alignment. Next, the orbicularis oculi muscle was repaired using buried interrupted 5-0 Vicryl ^®^ Rapid (Ethicon-Johnson & Johnson Medical Limited, Ohio, USA) sutures to restore muscular function and support. The eyelid margin was carefully approximated with two interrupted Nylon 5-0 stitches, which were left with long tails and suspended to the forehead with sterile tapes to provide postoperative support and maintain the correct position during the healing phase. In the upper eyelid, a laterally based orbicularis oculi myocutaneous flap was harvested (Figure 1c). Dissection of the lateral third of the flap was performed with great care to avoid injury to critical structures such as the lateral palpebral vessels and the marginal arcade. The flap was then transposed to the recipient area and fixed with Nylon 5-0 interrupted sutures (Figure 1d). The donor site in the upper eyelid was primarily closed with Nylon 5-0 interrupted sutures (Figure 1d).

At the conclusion of the surgery, Tobramycin 0.3% antibiotic ointment was applied to the conjunctival sac to reduce the risk of infection, and the eye was patched for protection and comfort. Postoperative care included prescribing antibiotic eye drops and ointment (Tobramycin 0.3%) along with systemic analgesics for a duration of two weeks to manage pain and prevent infection. Lubricants were also prescribed for application three times daily to maintain ocular surface hydration. Skin sutures were removed within 5–7 days, while tarsal and marginal eyelid sutures were removed after 7–10 days, based on the assessment of wound healing progress.

### 2.2. Statistical Analysis

The collected data were entered in a Microsoft Excel sheet 2021 for analysis. Continuous variables were summarized and presented using the median value alongside the range. Discrete variables were analyzed and expressed as percentages.

## 3. Results

A total of 36 patients, comprising 33 males and 3 females, were included in this study to analyze the outcomes of the three-step surgical technique for the correction of involutional lower eyelid ectropion The demographic and clinical characteristics of these patients are presented in detail in Table 1. Preoperative symptoms reported by the patients included persistent epiphora, characterized by excessive tearing due to improper tear drainage, ocular pain resulting from the exposure of sensitive ocular surfaces, and dry eye syndrome caused by the inability of the eyelid to maintain adequate contact with the ocular surface.

**Figure 2:**
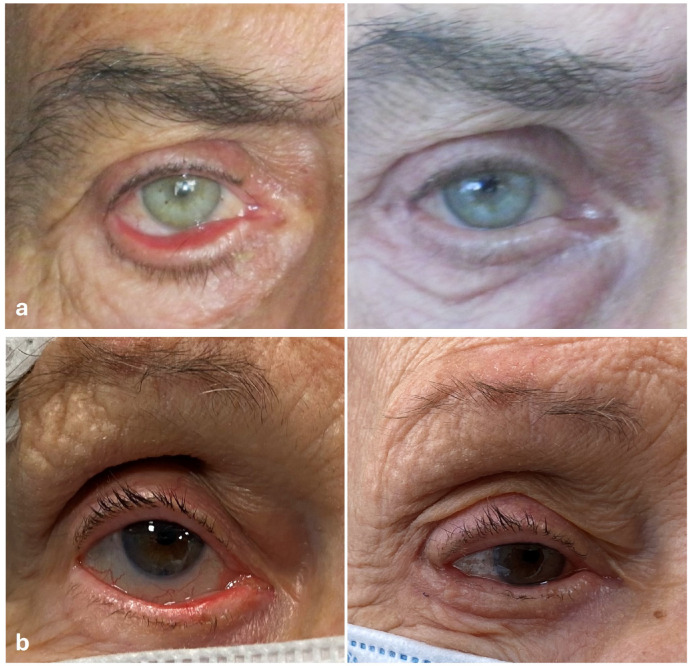
(**a**) Seventy-three-years-old male patient with involutional ectropion of the right lower eyelid. On the left, the pre-operative picture showing a grade III ectropion with exposure of the lower eyelid fornix; on the right, the postoperative picture showing a normal apposition of the lower eyelid to the ocular surface, taken 12 months after surgery; (**b**) Eighty-one-years-old female patient with involutional ectropion of the right lower lid. On the left, the pre-operative picture showing a grade II ectropion with the exposure of the lower eyelid conjunctiva; on the right, the postoperative picture showing a normal apposition of the lower eyelid to the ocular surface, taken 12 months after surgery.

In our study group, there were no cases of immediate postoperative complications, such as hematoma or infection. However, one case of early wound dehiscence was reported, specifically involving the tarsectomy incision. This dehiscence occurred immediately following the removal of stitches after seven days. To address this issue, the stitches were repositioned, and their removal was deferred until 10 days post-surgery, successfully resolving the dehiscence without further complications. At three months follow-up, optimal eyelid position was observed in 42 (93.3%) cases, while 2 (4.4%) patients had a residual scleral show but did not exhibit any inflammatory eye symptoms and thus did not require additional surgical intervention. All patients, including those with residual scleral show, expressed satisfaction with both the functional and esthetic outcomes of the procedure (Figure 2). One case of undercorrection was identified, requiring surgical revision involving additional eyelid shortening to achieve the desired eyelid position. Following this revision, the patient achieved a satisfactory outcome. Additionally, preoperative symptoms were resolved in all other patients. No cases of late complications, such as scar contracture, lid margin peaking, overcorrection resulting in entropion, or trichiasis, were reported. At three-year follow-up, two cases (4.4%) of grade II ectropion recurrence were detected. These cases were effectively managed with adjunctive eyelid shortening using a wedge resection, yielding satisfactory results.

## 4. Discussion

The surgical management of involutional ectropion aims to restore the normal eyelid position to ensure complete lid closure and adequate corneal protection. Achieving the successful correction of ectropion depends on a thorough comprehension of eyelid mechanics, and this understanding serves as the foundation for devising effective surgical strategies. Since eyelid malposition usually stems from eyelid laxity and mechanical imbalance between the anterior and posterior lamellae, surgical intervention should be designed to address both etiological factors systematically.

The increase in the horizontal length of the eyelid is recognized as the primary factor in the pathogenesis of involutional lower eyelid ectropion. This elongation contributes to the loss of contact between the eyelid and the eyeball, ultimately allowing the eyelid to rotate outward, which results in ectropion [6]. To address this core issue, the main step of our combined surgical procedure involves eyelid shortening by removing a tarso-conjunctival base-up triangle, a modification of Bick’s technique [9]. This precise triangular resection serves to invert the upper portion of the tarsus backward against the globe while simultaneously pulling it upward. Together, these adjustments effectively restore the normal lid margin position and improve eyelid stability [18]. The upward and inward tension generated by shortening the eyelid is instrumental in counteracting both vertical descent and horizontal eversion, thereby restoring the lower eyelid’s natural position and function. Among alternative techniques for correcting horizontal lower eyelid laxity, the lateral tarsal strip (LTS) procedure [10,19,20] has been widely documented in the literature. Both the wedge resection and LTS have proven to be effective and reliable techniques for tightening the eyelid [13,18,21,22,23]. However, there are critical differences between these two techniques. First, the lateral incision required in the LTS technique can potentially damage the integrity of the lateral canthal tendon (LCT), increasing the risk of complications, while the wedge resection avoids interferences with this structure completely. Second, the LTS technique involves burying the tarsal plate, which introduces the risk of granuloma formation at the lateral canthus, likely due to the presence of Meibomian glands within the buried tissue [24]. This complication is typically not observed with Bick’s procedure. One of the main criticisms of lateral wedge resection is the potential for blunting and medial displacement of the lateral canthal angle, which can compromise both function and esthetics [10,19,25]. However, none of the patients in our cohort experienced such deformity. This positive outcome is likely attributable to the preservation of both the LCT insertion and the lateral canthal angle during surgery in our modified technique. Regarding the rate of wound dehiscence, a recent paper by Vahdani et al. [23] compared the outcomes of wedge resection and the LTS procedure in treating lower eyelid malpositions. They reported a higher incidence of wound dehiscence and reoperation in the LTS group. In our case series, only one case of early wound dehiscence was observed, and this was most likely due to premature stitch removal rather than any inherent issue with the procedure itself. Taking all these factors into account, we consistently prefer the wedge resection technique over the LTS procedure for addressing lower eyelid laxity. This preference is informed by its simplicity, lower complication profile, and consistent results in restoring the natural position and function of the eyelid.

The second crucial step of our technique involves releasing the lower eyelid skin to mitigate the out-pulling vector caused by secondary inflammatory changes in the eyelid skin. Chronic epiphora, a common consequence of longstanding ectropion, often induces inflammation and subsequent contracture of the lower eyelid skin, exacerbating the lower lid malposition. Accurate skin release represents therefore an essential step in our procedure, as it allows the shortened eyelid to return to normal position without any impediment. At the same time, this procedure leads to a skin deficit of the anterior lamella that requires reconstruction.

The last step of our combined procedure therefore consists of the transposition of a laterally based myocutaneous flap from the upper eyelid to cover the skin deficit resulting from the previous step and stabilize the eyelid position. Skin grafting is another alternative method for managing anterior lamella skin deficits, but it often carries risks of graft rejection, scarring, and esthetic mismatches. The use of a laterally based orbicularis oculi myocutaneous flap, as outlined in this technique, instead, provides vascularized tissue for reconstruction, and additional support and suspension to the lower eyelid [26,27]. The anatomical orientation of the flap, in fact, ensures an upward pulling orientation of forces that counteract the forces contributing to ectropion [26,27]. Other advantages of using tissues from the upper lid are the low donor site morbidity with no loss of function and the good esthetic result, as the scar is always well-hidden in the supra-tarsal fold of the upper eyelid [26,27]. Even though one may argue about a potential asymmetry with the contralateral upper eyelid, in case of unilateral ectropion repair, none of the patients included in our cohort complained about this issue.

In our study, most of the patients were 70 years old or over. Males were predominant over females in this study, as in several other studies [22,28,29]. This male predominance has been previously attributed to anatomical differences, such as a larger tarsal plate in men than females [30]. A larger tarsus can facilitate the onset of ectropion as it mechanically adds an outpulling force to the balance of forces that maintain the eyelid in its position. In our cohort, unilateral ectropion was observed in 75% of cases, while bilateral involvement was less common, affecting 25% of patients.

Surgical complications in our series were minimal, with no major adverse events, such as hematoma or infection, demonstrating the safety of the surgical technique. Only one case of early wound dehiscence was reported, specifically involving the tarsectomy incision, related to a premature stitch removal; repositioning of the stitches was sufficient to resolve the dehiscence. At the three-month follow-up, an optimal eyelid position was observed in 93.3% of the cases, reflecting a high rate of success. Our results are comparable to the ones shown in other studies [13,22,23,28]. One case of undercorrection was identified, requiring surgical revision involving additional eyelid shortening to achieve the desired result. Additionally, preoperative symptoms were resolved in all other patients, highlighting the effectiveness of the procedure in alleviating discomfort and restoring functionality. No cases of adverse complications, such as scar contracture, lid margin peaking, overcorrection resulting in entropion, or trichiasis, were reported, further underscoring the safety of this technique. At the three-year follow-up, two cases of recurrence (grade II ectropion) were detected. These cases were effectively managed with adjunctive eyelid shortening using a wedge resection, yielding satisfactory results. The overall findings emphasize the reliability of this surgical approach, with minimal complications and high patient satisfaction rates.

While traditional techniques such as LTS and wedge resection have their merits, this detailed, multi-faceted surgical approach provides a robust solution for managing lower eyelid involutional ectropion. By simultaneously addressing multiple factors involved in ectropion development, it offers comprehensive correction with reduced risk of complications and recurrence. This makes it particularly advantageous for patients with moderate to severe ectropion, where single-technique approaches may fall short.

Our 3-step procedure is designed as an innovative approach that incorporates key principles from several well-known techniques for the treatment of involutional lower eyelid ectropion, combining them in a single comprehensive method. This procedure is specifically tailored to address the multifactorial nature of involutional ectropion. The first step focuses on correcting lower eyelid instability through a wedge resection procedure, which effectively shortens and tightens the lengthened eyelid. This step addresses the core structural issue of horizontal laxity. The second step aims to restore the balance between the anterior and posterior lamellae by releasing the skin to reduce the outward-pulling vector. This adjustment stabilizes the eyelid’s position and creates conditions that better support normal eyelid function. The third step involves an inverting procedure using a musculocutaneous flap harvested from the upper eyelid, which enhances the inward tension necessary for correcting eversion. The principles behind this surgical approach are highly versatile and can also be adapted to treat involutional entropion, as the two conditions are essentially mirror images of the other. By targeting the causative factors and underlying mechanisms, this technique provides complete, reliable, and lasting correction in treating grade II and III involutional lower eyelid ectropion. Additionally, the multi-step nature of the procedure allows for a nuanced approach, ensuring that both functional and esthetic outcomes are optimized. Our results underscore the safety and efficacy of this method. By addressing each contributory factor comprehensively, the 3-step procedure achieves a durable resolution of symptoms and malposition. We strongly believe this approach represents a robust surgical solution for treating moderate to severe cases of involutional ectropion, offering patients not only functional relief but also enhanced quality of life.

However, this study has limitations, including a small patient sample and its retrospective design, which lacked direct comparisons with other techniques. Future prospective studies with larger cohorts and longer follow-up periods are needed to validate these findings.

## 5. Conclusions

The novel 3-step surgical technique described in this study offers a safe and effective approach to correcting involutional lower eyelid ectropion. By addressing horizontal laxity, anterior lamellar deficiency, and gravitational forces, this procedure achieves durable functional and esthetic outcomes with minimal complications. Future research should focus on comparing this approach to other surgical techniques to better define the effectiveness and long-term outcomes.

## Figures and Tables

**Table 1:** Demographic data and clinical characteristics of the patients included in the study.

Variable		No.	Percentage
Age (years)	Median	77	
	Range	(55–92)	-
Gender	Male	33	92%
	Female	3	8%
Eyelid	Right	16	36%
	Left	11	24%
	Bilateral	9	40%
	Total	45	-
Degree of ectropion	Grade I	0	0%
	Grade II	32	29%
	Grade III	13	71%
Follow-up (months)	Mean	22	
	(Range)	(3–144)	

## Data Availability

The data used and/or analyzed during the current study are available from the corresponding author upon reasonable request.
